# Novel Species Diversity in China’s Northeastern Border Region

**DOI:** 10.3390/v18070735

**Published:** 2026-07-02

**Authors:** Linghui Yuan, Na Zhang, Min Yuan, Jianguo Xu, Zhiguo Liu, Zhenjun Li

**Affiliations:** 1 National Key Laboratory of Intelligent Tracking and Forecasting for Infectious Diseases, National Institute for Communicable Disease Control and Prevention, Chinese Center for Disease Control and Prevention, Beijing 102206, China; yuanlinghui0301@163.com (L.Y.); zzdr9906@163.com (N.Z.); yuanmin@icdc.cn (M.Y.); xujianguo@icdc.cn (J.X.); 2 School of Public Health, Inner Mongolia Medical University, Hohhot 010110, China

**Keywords:** northeastern border region of China, novel pathogens, reverse microbial etiology

## Abstract

The northeastern region of China is characterized by complex ecosystems, including forests and wetlands, and borders North Korea, Russia, and Mongolia. It serves not only as a natural reservoir for various microorganisms but also as a critical geographical and ecological hub for cross-border exchanges in Northeast Asia. Based on metagenomics and meta-transcriptomics investigations, this study systematically reviews the current research status of novel pathogens in the northeastern border region of China. It systematically organizes the newly discovered species, their classifications, and geographical distributions, with a focus on analyzing novel viruses that have potential pathogenicity to humans. The novel viruses identified in the northeastern border region belong to 11 viral families, including 9 from the *Nairoviridae*, 4 from the *Rhabdoviridae*, 3 each from the *Astroviridae*, *Picornaviridae*, and *Parvoviridae*, and 1–2 from other viral families, indicating a broad diversity of newly discovered viruses. These novel viruses are found in a wide range of hosts, including humans, ticks, minks, *Marmota sibirica*, and *Myodes rufocanus*, underscoring the significant public health risks these viruses pose. Geographically, the novel viruses discovered in the northeastern border region show a clustering pattern, with new species primarily concentrated in areas bordering Russia and North Korea. This highlights the unique role of the region as a hotspot for cross-border pathogen transmission and risk. The findings provide a systematic scientific reference for understanding the spectrum of unknown novel pathogens and their geographical distribution in the northeastern border region, assessing the risk of emerging infectious diseases, and optimizing active surveillance systems.

## 1. Introduction

In an increasingly interconnected world, the threat of pandemics posed by emerging infectious diseases has garnered growing recognition. In 2019, the concept of “reverse microbial etiology” was first proposed by Academician Xu Jianguo, and its core idea centered on transforming the traditional passive response model into proactive surveillance for the identification of potentially pathogenic microorganisms, thereby laying the foundation for the prediction and prevention of the next pandemic [1]. Guided by this concept, the systematic review and summary of novel pathogen discovery achievements in active surveillance practices have been considered of significant scientific value for defining the current boundaries of knowledge and optimizing future surveillance strategies. This study has focused on the northeastern border region of China, a high-risk area characterized by ecological complexity and frequent cross-border activities, and has aimed to comprehensively review the diversity and distribution characteristics of novel viruses proactively discovered through metagenomic and meta-transcriptomic technologies, so that a scientific basis may be provided for the active prevention and control of emerging infectious diseases in this region and other similar areas. The northeastern border region of China—comprising Heilongjiang, Jilin, and Liaoning provinces and the five eastern leagues of Inner Mongolia—borders North Korea, Russia, and Mongolia. This region, where forests, grasslands, and wetlands are integrated, has been recognized as a natural focus for diverse pathogens and has been characterized by ecological complexity and frequent cross-border activities. Among the pathogens known to be active on both sides of the border, Hantaan virus and Borrelia burgdorferi have been identified [2,3,4]. In recent years, a variety of pathogens, such as Yezo virus and Alongshan virus, have been discovered in this region, fully indicating that this area is a hotspot for the discovery of novel pathogens [5,6]. Therefore, the surveillance and exploration of potentially pathogenic microorganisms in the northeastern border region urgently need to be strengthened. Through the proactive exploration of pathogen diversity, the capacity for discovering novel bacteria and viruses has been greatly enhanced, which has provided significant strategic value for the early warning and proactive prevention of emerging infectious diseases [7]. This study aimed to systematically review the progress in the discovery of novel species through metagenomics and meta-transcriptomics in the northeastern region and its border areas, and thus provides a reference for strengthening the discovery of novel pathogens in border regions.

## 2. Materials and Methods

### 2.1. Literature Retrieval Strategy

To systematically investigate the current landscape of microbial etiology research utilizing metagenomic and meta-transcriptomic technologies in northeastern China, we conducted a comprehensive literature search in the Web of Science Core Collection and PubMed. The search was restricted to publications from database inception to 15 December 2025, with no language restrictions applied. The detailed search strings, combining MeSH terms and free-text keywords, are provided in Table 1. All retrieved records were imported into EndNote 21 (Clarivate, Philadelphia, PA, USA; version 21.0) for deduplication and further screening.

The inclusion criteria were: (1) original research articles reporting the discovery of novel microbial species or novel viruses; (2) studies that applied metagenomic or meta-transcriptomic sequencing as one of the primary detection methods; and (3) studies with sample origins explicitly located in northeastern China (Liaoning, Jilin, Heilongjiang, and eastern Inner Mongolia).

The exclusion criteria comprised: (1) non-original literature (e.g., reviews, editorials, conference abstracts, news, patents, and standards); (2) duplicate publications, where only the most complete or most recent report was retained; and (3) studies that failed to provide any phylogenetic analysis or taxonomic basis for the novel species/virus designation.

### 2.2. Literature Data Extraction

Given the heterogeneity in sequencing strategies, assembly methods, and taxonomic validation approaches across studies, we performed a descriptive quality assessment of all included literature based on the information reported in the original articles. Since the primary studies generally did not report uniform quantitative metrics, such as genome completeness, sequencing coverage, or average nucleotide identity (ANI), we did not apply uniform numerical cutoffs as screening thresholds. Instead, we constructed a tiered evaluation framework based on objectively extractable information directly available from the literature, with reference to the practices of the International Committee on Taxonomy of Viruses (ICTV) and prokaryotic nomenclature codes.

Specifically, for each included article, reviewers extracted and recorded the following directly discernible information: (a) sequencing type (metagenomics or meta-transcriptomics); (b) genome acquisition status—whether the study obtained a complete genome sequence, multiple gene segments (e.g., two or more of the viral L/M/S segments), or a single short gene fragment (e.g., partial 16S rRNA or a single functional gene) for the novel species/virus; (c) phylogenetic analysis methods and the sequence regions on which they were based; (d) whether the taxonomic position of the species/virus received statistical support in the phylogenetic tree (e.g., bootstrap values); (e) whether the nomenclature of the novel species/virus followed international naming standards (e.g., ICNP or ICTV criteria); and (f) whether virus/pathogen isolation was successfully performed.

Based on the above information, we categorized the strength of evidence supporting each novel species/virus into three tiers. High confidence was assigned to studies that obtained the complete genome sequence of the novel species/virus, constructed phylogenetic trees based on the complete genome or multiple complete gene segments with high statistical support (e.g., bootstrap values ≥ 70%), complied with international nomenclature standards (e.g., ICTV or ICNP criteria), and successfully performed virus isolation. Moderate confidence was assigned to studies that obtained the complete genome sequence and fulfilled the phylogenetic and nomenclature criteria described above, but lacked virus isolation validation. Low confidence was assigned to studies that obtained multiple gene segments (e.g., two or three viral genome segments) and performed phylogenetic analysis but did not provide a complete genome sequence; to studies that relied solely on a single short fragment sequence (e.g., partial 16S rRNA, a single functional gene, or NGS short reads) for species/virus identification; or to studies with low phylogenetic support (bootstrap values < 70%) or those that did not clearly provide taxonomic naming justification. This tiered classification was used not as an exclusion criterion but as an analytical framework to contextualize the reliability of the findings during subsequent synthesis and interpretation. All classification results were cross-verified by two independent reviewers, with discrepancies resolved through consensus.

From the studies that met the inclusion criteria, we extracted the following core data using a pre-designed standardized form: (a) novel species/virus name (or provisional designation; if unnamed, the designation used in the original article was adopted); (b) taxonomic classification (family/genus); (c) year of first report; (d) genome acquisition status (complete genome/multiple gene segments/single gene fragment); (e) host species or sample type from which the species/virus was first detected; (f) geographic information of sample collection (specific coordinates or administrative regions); and (g) whether virus isolation was successful. For novel species/viruses with documented human pathogenicity, we additionally extracted the following: (a) known geographical distribution; (b) prevalence or infection rates; (c) associated clinical manifestations; and (d) documented or suspected primary vectors/reservoirs.

## 3. Results

### 3.1. Novel Species in the Northeastern Region

Through systematic retrieval and manual supplementation, 117 articles were initially obtained in this study. After 39 duplicate articles were removed by EndNote software, 78 articles remained. By browsing the titles and abstracts, 34 articles that were irrelevant or did not meet the inclusion criteria were excluded. The remaining 44 articles were read in full text, and 12 articles that met the criteria were finally included. The publication dates of the articles were concentrated between 2019 and 2025. In the 12 articles finally included, a total of 30 novel virus species were reported. Based on the confidence classification criteria described in Section 2.2, we systematically classified the 30 novel viruses identified in the included studies. High-confidence viruses comprised 5 species, including Wetland virus, Xue-Cheng virus, Songling virus, Antu virus and Dadong virus. These studies all obtained complete genome sequences, constructed phylogenetic trees with high statistical support, and complied with International Committee on Taxonomy of Viruses (ICTV) nomenclature standards, with most successfully achieving virus isolation. The moderate-confidence viruses, which comprised 11 species, was characterized by the acquisition of multiple gene segments or complete genomes but lacked virus isolation validation. Low-confidence viruses comprised 14 species, including eight mink stool-associated viruses, four Chenqi marmot-associated viruses, as well as Jiutai virus and Jilin tick virus 1, all of which were represented only by partial genomic fragments without complete genome assembly or viral isolation.

All these viruses belonged to the following 11 viral families: 9 species were from the *Nairoviridae*; 4 species were from the *Rhabdoviridae*; 3 species each were from the *Astroviridae*, *Picornaviridae*, and *Parvoviridae*; 2 species each were from the Flaviviridae and *Caliciviridae*; and 1 species each was from the *Filoviridae*, *Phenuiviridae*, *Chuviridae*, and *Tombusviridae*. The host range of the novel viruses was found to be broad, including humans, ticks, minks, *Marmota sibirica*, and *Myodes rufocanus* (Table 2). These viruses constitute a potential pathogen reservoir for local emerging infectious diseases, and among the 30 novel species, 4 species have been confirmed to be pathogenic to humans.

### 3.2. Virus Species with Potential Pathogenic Risk

Among the numerous novel species, the identification of viruses with potential public health risks has been considered key to assessing the threat of emerging infectious diseases and guiding active surveillance. Among the 30 newly discovered virus species, 4 tick-borne viruses belonging to *Nairoviridae* were found to be associated with human infection, namely Beiji nairovirus, Songling virus, Wetland virus, and Xue-cheng virus. Among these, Songling virus, Wetland virus, and Xue-cheng virus were successfully isolated, or their nucleic acids were detected directly from blood samples of patients with a history of tick bites, thus providing direct molecular evidence for infection in humans. Beiji nairovirus was first identified from ticks and was later successfully isolated from blood samples of patients with a history of tick bites (Table 3).

#### 3.2.1. Beiji Nairovirus

Beiji nairovirus (BJNV) is a virus belonging to the family *Nairoviridae* (genus Nairovirus), and it was the first virus of the genus *Nairovirus* worldwide to be unequivocally confirmed to infect humans. In 2019, BJNV was first discovered in *I. persulcatus* in Heilongjiang Province, China, and was later detected in the blood of hospitalized patients with a history of tick bites, from whom the complete genome of BJNV was obtained [8]. The clinical symptoms of the patients included non-specific symptoms such as fever, headache, and malaise, and fatal cases have also been reported [20]. The discovery process of Beiji nairovirus—first identified in ticks and later confirmed to be pathogenic to humans—has strongly demonstrated the critical role of metagenomic sequencing and reverse microbial etiology in the discovery of modern emerging infectious diseases.

#### 3.2.2. Songling Virus

Songling virus (SGLV) was first isolated and identified from the serum of patients with a history of tick bites in Heilongjiang Province in 2021, and it is a novel pathogen of the genus *Orthonairovirus* that can infect humans [10]. Subsequently, human infection cases were also found in the Inner Mongolia Autonomous Region. More importantly, antibodies against Songling virus were detected in animal sera in the Xinjiang Uygur Autonomous Region, which strongly suggested that an animal infection cycle of this virus has already existed over a broader geographical range, posing a potential regional epidemic risk [21]. The clinical manifestations in patients infected with Songling virus included non-specific symptoms such as headache, fever, depression, fatigue, and dizziness. Serological studies indicated that virus-specific antibody responses could be produced in more than two-thirds of the patients during the acute phase [10].

#### 3.2.3. Wetland Viruses

Wetland virus (WELV) was first reported in 2024. It was first identified in a patient who developed persistent fever and multiple organ dysfunction after a tick bite in a wetland park in the Inner Mongolia Autonomous Region in June 2019, and it was detected in the patient’s serum by metagenomic sequencing. Through subsequent active surveillance, multiple infection cases were confirmed among febrile patients in Inner Mongolia, Heilongjiang, Jilin, and Liaoning. Wetland virus was detected in humans, five tick species (especially *H. concinna*), and various animals (such as sheep, horses, pigs, and *Myospalax psilurus*) in the northeastern region of China, which suggested that it has a broad host infection spectrum and a complex natural circulation. The clinical manifestations after human infection were diverse, and the most common symptoms included fever, dizziness, headache, and fatigue. Notably, petechiae and lymphadenopathy were observed in some patients, and neurological manifestations (such as irritability, lethargy, and coma) were presented in individual cases [14].

#### 3.2.4. Xue-Cheng Virus

Xue-Cheng virus (XCV) was first reported in 2025 and was discovered by a research team through meta-transcriptomic sequencing of sera from 252 febrile patients with a history of tick bites at a sentinel hospital in Mudanjiang City, Heilongjiang Province, between May and July 2023. Xue-Cheng virus was detected in both *H. concinna* and *H. japonica*, suggesting that these two tick species may be its transmission vectors. Through RT-PCR testing of sera from 792 participants between 2022 and 2024, a total of 26 infected individuals were identified, with an infection rate of 3.3%. The clinical manifestations of the infected individuals ranged from non-specific acute febrile illness to severe disease requiring hospitalization [15].

These viruses are all tick-borne pathogens. The post-infection manifestations are predominantly non-specific symptoms, including fever, headache, and fatigue, and are frequently accompanied by laboratory abnormalities such as leukopenia and thrombocytopenia. Consequently, these manifestations are easily confused with those of other common tick-borne diseases in clinical practice. Their successful identification was achieved through the combination of next-generation sequencing technology and active clinical sentinel surveillance, and these have been considered important practical achievements guided by the reverse microbial etiology strategy.

### 3.3. Ecological Significance of Novel Viruses Identified in Animals

Among the 30 newly discovered virus species, apart from the 4 tick-borne viruses that have been confirmed to be pathogenic to humans, the remaining 26 viruses were first identified in animal hosts such as ticks, minks, *Marmota sibirica*, and *Myodes rufocanus*. Although these viruses have not yet been found to be directly associated with human diseases, their virological and ecological significance, from the One Health perspective, should not be overlooked [22].

First, novel virus species belonging to multiple viral families, including *Astroviridae*, *Picornaviridae*, and *Caliciviridae*, were identified in minks in Liaoning Province. It is noteworthy that several known members of the *Astroviridae* families have been documented to infect humans and cause diseases [23]. As economically important animals raised in large-scale farming systems, minks are in frequent contact with humans, and the novel viruses they carry have thus constituted a potential risk of cross-species transmission [24]. Second, *Marmota sibirica* is widely distributed in the forest and grassland ecosystems of the northeastern region, and their population dynamics, migratory behaviors, and occasional contact with livestock and humans may all influence the ecological circulation and spillover risk of these viruses [25,26]. Furthermore, although no evidence of human infection has been found for the non-pathogenic novel viruses identified in ticks at the present stage, their presence has enriched our understanding of the diversity of the tick virome and has provided baseline data for future surveillance of potential cross-species adaptation and pathogenic evolution of viruses.

In summary, sustained surveillance and in-depth investigation of animal-derived novel viruses have been considered not only an essential step for comprehensively assessing regional pathogen diversity, but also a critical measure for identifying potential zoonotic risks and achieving the transition from “passive response” to “proactive prevention.”

### 3.4. Geographical Distribution of Novel Viruses

**Figure 1:**
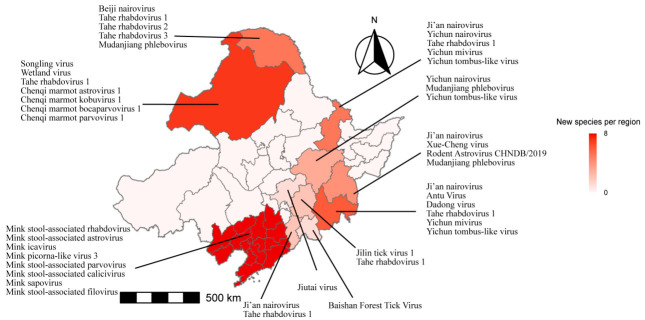
Geographical distribution of new species. Note: Base map sourced from the National Platform for Common Geospatial Information Services (Tianditu, https://www.tianditu.gov.cn (accessed on 5 June 2025)), under the map review number GS (2024) 0650.

From the perspective of provincial-level administrative divisions, differences were observed in the coverage of cities and prefectures where novel viruses were reported among the provinces (Figure 1). Heilongjiang Province has jurisdiction over 13 prefecture-level administrative divisions, including 12 prefecture-level cities and 1 prefecture. Areas with reports of novel pathogens involved 4 prefecture-level divisions, namely Da Hinggan Ling (Mohe, Tahe), Yichun, Mudanjiang (including Hailin), and Harbin, with a coverage rate of 30.77%. Among the 9 prefecture-level administrative units (including 1 autonomous prefecture) in Jilin Province, novel pathogens were reported in 5 areas, namely Yanbian Korean Autonomous Prefecture (Dunhua, Shulan, Antu, Helong), Baishan, Tonghua (Ji’an), Jilin (Jiaohe, Shulan), and Changchun (Jiutai), with a coverage rate of 55.56%. In Liaoning Province, only the provincial level (Liaoning) was indicated, without specification to the city or prefecture level. Among the five eastern leagues of the Inner Mongolia Autonomous Region, areas with definite reports of novel pathogens were Hulunbuir (Songling, Yakeshi), with a coverage rate of approximately 20%.

The surveillance coverage at the provincial and prefectural levels varied considerably. Jilin Province showed the highest coverage, especially in the Yanbian Korean Autonomous Prefecture (where reports involved 4 counties and cities), revealing a broad geographical distribution of novel species in this province. In Heilongjiang Province, risks were highly concentrated in key ecological areas. The 4 prefectures and cities involved in the reports (such as Da Hinggan Ling Prefecture, Yichun, and Mudanjiang) were, without exception, located in the core areas of major forest regions such as the Greater and Lesser Khingan ranges and the Changbai Mountains. This indicated that the risks in this province were strongly associated with forest ecosystems, with a clear spatial orientation. The data from Inner Mongolia and Liaoning revealed an imbalance in surveillance: in Inner Mongolia, the reports were currently precisely located to Hulunbuir (with a coverage rate of approximately 20%), which constitutes the main part of the Greater Khingan forest region. In Liaoning Province, only the provincial level was indicated, and the granularity of the reported data was relatively coarse, potentially underestimating or obscuring the actual risk points.

**Figure 2:**
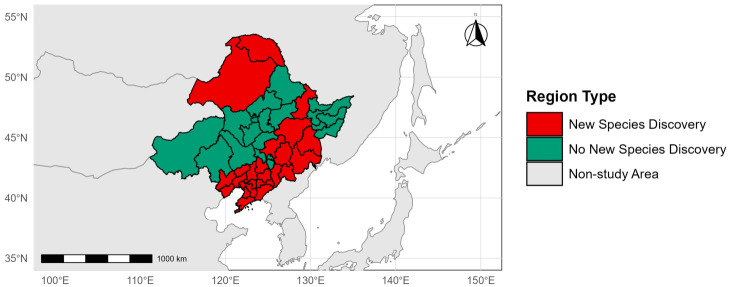
Comparative Analysis of Border Regions. Note: Base map sourced from the National Platform for Common Geospatial Information Services (Tianditu, https://www.tianditu.gov.cn (accessed on 5 June 2025)), under the map review number GS (2024) 0650.

Figure 2 illustrates the geographic distribution of the novel species/viruses included in this study. The discovery sites of all 30 novel viruses could be classified into two major geographic categories: (1) Core ecological risk areas—major mountain forest regions: The three major mountain systems, namely the Greater Khingan Mountains, the Lesser Khingan Mountains, and the Changbai Mountains, encompassed nearly all the discovery sites in Heilongjiang, Jilin, and Inner Mongolia. These continuous and intact forest ecosystems provide an ideal ecological environment for hosts such as ticks, and have been considered source risk areas for the natural circulation of viruses. (2) Border areas—border ports and their radiation zones: The reported sites, such as the Yanbian Korean Autonomous Prefecture (the border area of China, North Korea, and Russia), Hulunbuir (the border area of China, Russia, and Mongolia), and Mudanjiang (a port city to Russia), were also locations of important land border ports. These areas carry dual risks: they are not only areas of local ecological risk but also potential entry points for exotic pathogens through cross-border commercial activities, personnel movements, or vector migration.

## 4. Discussion

The northeastern region of China, as a border area adjacent to Russia, North Korea, and Mongolia, has been considered a high-risk area for cross-border transmission of infectious diseases due to its unique ecological environment, intensive trade activities, and wildlife migration routes. In 1910, pneumonic plague was introduced along the Siberia–Manchuria–Harbin railway route. The outbreak was concentrated in Harbin and rapidly spread along the railway to the entire northeastern and northern regions of China, eventually resulting in more than 60,000 deaths [27]. In 2018, African swine fever was introduced from the Russian Far East through Shenyang, Liaoning Province. After the outbreak was first reported in the northeastern region, it rapidly spread across the country through the domestic transportation of live pigs, causing heavy losses to the Chinese pig industry [28]. Based on the above historical lessons and the findings of this study, the following active surveillance and prevention measures should be implemented in the identified high-risk ecological units. First, in the core forest ecosystems (the Greater Khingan Mountains, the Lesser Khingan Mountains, and the Changbai Mountains) and the surrounding agro-pastoral ecotones, a routine veterinary surveillance network covering livestock and farmed animals (such as cattle, sheep, pigs, and minks) should be established, and serum and tissue samples should be regularly collected for metagenomic screening, so that early warning of potential zoonotic viruses can be achieved. Second, in border ports and their radiation zones (such as the Yanbian Korean Autonomous Prefecture, Hulunbuir, and Mudanjiang), quarantine and pathogen surveillance of cross-border transported animals and animal products should be strengthened to prevent the importation of exotic pathogens through trade or animal migration routes. Third, in high-risk areas where wild rodents such as *Marmota sibirica* and *Myodes rufocanus* are distributed, long-term longitudinal surveillance of wildlife pathogen ecology should be carried out to systematically assess seasonal fluctuation patterns of carried viruses, host population dynamics, and cross-species transmission risks. Through the whole-chain active surveillance, the key nodes of cross-species transmission are expected to be identified and interrupted before outbreaks spill over into human populations, thereby truly translating the “reverse microbial etiology” concept into operational, regional biosecurity prevention and control practices.

In this study, novel pathogens discovered through metagenomics or meta-transcriptomics in the northeastern border region of China were investigated for the first time. As of 2025, this study included 12 studies on reverse microbial etiology discovery based on the above-mentioned technologies in the northeastern region, which cumulatively reported 30 newly discovered viruses. Meanwhile, the 30 newly discovered virus species were not evenly distributed among the studies; rather, several independent investigations achieved the discovery of multiple novel species across multiple regions within a single study. Among the 12 studies finally included, one-third (4 studies) reported the discovery of two or more novel virus species in a single study. This pattern was reflected in samples of different host types: among the 5 studies in which multiple viruses were discovered, 2 studies were based on tick samples, in which 2 and 8 novel viruses were discovered, respectively; similar findings were also observed in studies on other animals, with one study on minks reporting 8 novel viruses and another on marmots reporting 4. This phenomenon has indicated that the technology can efficiently and broadly reveal multiple unknown viruses within the same ecological environment or host group in a single study, thereby significantly enhancing the efficiency and coverage of novel pathogen discovery and providing more proactive and forward-looking technical support for the early warning and prevention and control of infectious diseases.

However, certain limitations were revealed by our data. During the data analysis, it was found that a small number of high-yield publications exerted a decisive influence on the number of reports of novel viruses at the provincial and even prefectural levels. For example, only one publication was retrieved for Liaoning Province, and 8 novel species were discovered in this high-yield publication. Secondly, because the existing data may have been influenced by research hotspot areas, the richness of novel species and pathogen diversity may have been underestimated, and the true regional situation may not yet have been fully represented. Finally, for the vast majority of novel viruses, only genome identification has been completed, and their key biological characteristics, such as pathogenicity, transmissibility, and host range, remain unknown, and a huge gap has thus existed from sequence discovery to risk confirmation.

In summary, the diversity, host range, and geographical distribution patterns of novel viruses in the northeastern border region of China have been systematically revealed in this study, and the core forest ecosystems and border port areas have been identified as two key types of high-risk geographical units for future novel pathogen discovery and surveillance. These findings have not only provided a scientific basis for the biosecurity defense of the northeastern region, but have also had universal implications beyond this region: against the backdrop of globalization and the persistent risk of emerging infectious diseases, only by proactively deploying systematic surveillance networks for unknown pathogens in similar high-risk areas, and by shifting the surveillance frontier from clinical patients to animal vectors, farmed animals, and wildlife, can the fundamental transition from “passive response” to “proactive prevention” truly be achieved. Active detection, proactive identification, and forward deployment of surveillance should be regarded as the core strategies for future regional biosecurity and the prevention and control of emerging infectious diseases.

## Figures and Tables

**Table 1:** Electronic search strategies for Web of Science and PubMed.

Database	Search Strategy
Web of Science	1. (metagenom* OR metatranscriptom* OR “next generation sequencing” OR NGS OR “high-throughput sequencing” OR “shotgun sequencing” OR mNGS OR “clinical metagenomics”) [Topic] 2. (China OR Chinese) [Topic] 3. (northeast* OR “north eastern” OR Heilongjiang OR Jilin OR Liaoning OR “Inner Mongolia” OR “border region*” OR frontier OR Russia OR Russian OR “North Korea” OR Mongolia OR Mongolian) [Topic] 4. (outbreak OR surveillance OR monitor* OR “emerging pathogen” OR “pathogen discovery” OR “unknown etiology” OR “fever of unknown origin” OR zoono OR “vector-borne” OR “virus discovery” OR “bacteria discovery” OR “One Health”) [Topic] 5. 1 AND 2 AND 3 AND 4
PubMed	1.”Metagenomics” [MeSH Terms] OR “Sequence Analysis, RNA” [MeSH Terms] OR “Disease Outbreaks” [MeSH Terms] OR “Communicable Diseases, Emerging” [MeSH Terms] OR “Public Health Surveillance” [MeSH Terms] OR “Sentinel Surveillance” [MeSH Terms] 2. (metagenom* OR metatranscriptom* OR “next generation sequencing” OR NGS OR “high-throughput sequencing” OR “shotgun sequencing” OR mNGS OR “clinical metagenomics”) [All Fields] 3. 1 OR 2 4. (China OR Chinese) AND (northeast* OR “north eastern” OR Heilongjiang OR Jilin OR Liaoning OR “Inner Mongolia” OR “border region*” OR frontier OR Russia OR Russian OR “North Korea” OR Mongolia OR Mongolian) [All Fields] 5. (“One Health” OR outbreak OR surveillance OR monitor* OR “emerging pathogen” OR “pathogen discovery” OR “unknown etiology” OR “fever of unknown origin” OR zoono OR “vector-borne” OR “virus discovery” OR “bacteria discovery”) [All Fields] 6.3 AND 4 AND 5

Note: Asterisk (*) indicates a truncation symbol (right-hand wildcard) that enables retrieval of all morphological variants sharing the same word stem. For instance, the search term “metagenome *” captures “metagenome,” “metagenomic,” “metagenomics,” and other derivations thereof.

**Table 2:** List and distribution of newly described species.

Viral Family	Virus Name	Region of First Discovery	Year of First Report	Detection Method	Sample Source of First Identification	Reference
*Nairoviridae*	Beiji nairovirus	Mohe, Da Hinggan Ling Prefecture, Heilongjiang Province	2019	Metagenomics	Human (tick-bite history)	[8]
	Jilin Tick virus 1	Jiaohe, Jilin City, Jilin Province	2019	Metagenomics	Tick	[9]
	Songling virus	Songling, Hulunbuir City, Inner Mongolia Autonomous Region	2021	Metagenomics	Human (tick-bite history)	[10]
	Ji’an nairovirus	Yichun and Mudanjiang, Heilongjiang Province; Dunhua, Yanbian Korean Autonomous Prefecture, and Ji’an, Tonghua City, Jilin Province	2022	Meta-transcriptomics	Tick	[11]
	Yichun nairovirus	Yichun and Fangzheng County, Harbin City, Heilongjiang Province	2022	Meta-transcriptomics	Tick	[11]
	Antu Virus	Antu County, Yanbian Korean Autonomous Prefecture, Jilin Province	2023	Metagenomics	Tick	[12]
	Dadong virus	Dadong Village, Helong City, Yanbian Korean Autonomous Prefecture, Jilin Province	2024	Meta-transcriptomics	Tick	[13]
	Wetland virus	Yakeshi, Hulunbuir City, Inner Mongolia Autonomous Region	2025	Metagenomics	Human (tick-bite history)	[14]
	Xue-Cheng virus	Mudanjiang, Heilongjiang Province	2025	Meta-transcriptomics	Human (tick-bite history)	[15]
*Rhabdoviridae*	Tahe rhabdovirus 1	Tahe County and Yichun, Da Hinggan Ling Prefecture, Heilongjiang Province; Songling, Hulunbuir City, Inner Mongolia Autonomous Region; Dunhua, Yanbian Korean Autonomous Prefecture, Shulan, Jilin City, and Ji’an, Tonghua City, Jilin Province	2022	Meta-transcriptomics	Tick	[11]
	Tahe rhabdovirus 2	Tahe County, Da Hinggan Ling Prefecture, Heilongjiang Province	2022	Meta-transcriptomics	Tick	[11]
	Tahe rhabdovirus 3	Tahe County, Da Hinggan Ling Prefecture, Heilongjiang Province	2022	Meta-transcriptomics	Tick	[11]
	Mink stool-associated rhabdovirus	Liaoning Province	2025	Meta-transcriptomics	Mink	[16]
*Astroviridae*	Rodent Astrovirus CHNDB/2019	Hengdaohezi Town, Hailin City, Mudanjiang, Heilongjiang Province	2022	Metagenomics	*Myodes rufocanus*	[17]
	Mink stool-associated astrovirus	Liaoning Province	2025	Meta-transcriptomics	Mink	[16]
	Chenqi marmot astrovirus 1	Hulunbuir City, Inner Mongolia Autonomous Region	2025	Meta-transcriptomics	Marmot	[18]
*Picornaviridae*	Mink icavirus	Liaoning Province	2025	Meta-transcriptomics	Mink	[16]
	Mink picorna-like virus 3	Liaoning Province	2025	Meta-transcriptomics	Mink	[16]
	Chenqi marmot kobuvirus 1	Hulunbuir City, Inner Mongolia Autonomous Region	2025	Meta-transcriptomics	Marmot	[18]
*Parvoviridae*	Mink stool-associated parvovirus	Liaoning Province	2025	Meta-transcriptomics	Mink	[16]
	Chenqi marmot bocaparvovirus 1	Hulunbuir City, Inner Mongolia Autonomous Region	2025	Meta-transcriptomics	Marmot	[18]
	Chenqi marmot parvovirus 1	Hulunbuir City, Inner Mongolia Autonomous Region	2025	Meta-transcriptomics	Marmot	[18]
*Flaviviridae*	Jiutai virus	Jiutai District, Changchun City, Jilin Province	2019	Metagenomics	Tick	[9]
	Baishan Forest Tick Virus (BSFTV)	Baishan City, Jilin Province	2024	Meta-transcriptomics	Tick	[19]
*Caliciviridae*	Mink stool-associated calicivirus	Liaoning Province	2025	Meta-transcriptomics	Mink	[16]
	Mink sapovirus	Liaoning Province	2025	Meta-transcriptomics	Mink	[16]
*Phenuiviridae*	Mudanjiang phlebovirus	Fangzheng County, Harbin City, and Mudanjiang, Heilongjiang Province; Inner Mongolia Autonomous Region (Da Hinggan Ling Prefecture and surrounding areas)	2022	Meta-transcriptomics	Tick	[11]
*Chuviridae*	Yichun mivirus	Yichun, Heilongjiang Province; Dunhua, Yanbian Korean Autonomous Prefecture, Jilin Province	2022	Meta-transcriptomics	Tick	[11]
*Tombusviridae*	Yichun tombus-like virus	Yichun and Fangzheng County, Harbin City, Heilongjiang Province; Dunhua, Yanbian Korean Autonomous Prefecture, Jilin Province	2022	Meta-transcriptomics	Tick	[11]
*Filoviridae*	Mink stool-associated filovirus	Liaoning Province	2025	Meta-transcriptomics	Mink	[16]

**Table 3:** Emerging Tick-Borne Human Pathogenic Viruses in Northeast China.

Virus Name	Distribution Area	Infection Rate	Clinical Symptoms	Vector	References
Beiji nairovirus	Inner Mongolia Autonomous Region, Heilongjiang Province, Jilin Province	19.6% (129/658)	Fever, headache, malaise, coma, fatigue, myalgia, arthralgia, anorexia, rash, petechiae	*Ixodes persulcatus*, *Ixodes crenulatus*, *Dermacentor silvarum*, *Haemaphysalis longicornis*, *Haemaphysalis concinna*, *Dermacentor nuttalli*	[8,20]
Songling virus	Heilongjiang Province, Inner Mongolia Autonomous Region, Xinjiang Uygur Autonomous Region	6.4% (42/658)	Fever, headache, dizziness, fatigue, depression, rash, petechiae	*Ixodes crenulatus*, *Ixodes persulcatus*, *Haemaphysalis longicornis*, *Haemaphysalis concinna*	[10]
Wetland virus	Inner Mongolia Autonomous Region, Heilongjiang Province, Jilin Province, Liaoning Province	2.5% (17/682)	Fever, headache, dizziness, fatigue, lymphadenopathy, rash, petechiae	*Haemaphysalis concinna*, *Haemaphysalis longicornis*	[14]
Xue-Cheng virus	Heilongjiang Province	3.3% (26/792)	Fever, headache, dizziness, fatigue, lymphadenopathy, rash, petechiae	*Haemaphysalis concinna*, *Haemaphysalis japonica*	[15]

## Data Availability

No new data were created or analyzed in this study.
